# The Role of Phospholipase C in GABAergic Inhibition and Its Relevance to Epilepsy

**DOI:** 10.3390/ijms22063149

**Published:** 2021-03-19

**Authors:** Hye Yun Kim, Pann-Ghill Suh, Jae-Ick Kim

**Affiliations:** 1 Department of Biological Sciences, Ulsan National Institute of Science and Technology (UNIST), Ulsan 44919, Korea; yoon@unist.ac.kr (H.Y.K.); pgsuh@kbri.re.kr (P.-G.S.); 2 Korea Brain Research Institute (KBRI), Daegu 41062, Korea

**Keywords:** Phospholipase C (PLC), γ-aminobutyric acid (GABA), excitatory/inhibitory balance (E/I balance), GABAergic inhibition, epilepsy

## Abstract

Epilepsy is characterized by recurrent seizures due to abnormal hyperexcitation of neurons. Recent studies have suggested that the imbalance of excitation and inhibition (E/I) in the central nervous system is closely implicated in the etiology of epilepsy. In the brain, GABA is a major inhibitory neurotransmitter and plays a pivotal role in maintaining E/I balance. As such, altered GABAergic inhibition can lead to severe E/I imbalance, consequently resulting in excessive and hypersynchronous neuronal activity as in epilepsy. Phospholipase C (PLC) is a key enzyme in the intracellular signaling pathway and regulates various neuronal functions including neuronal development, synaptic transmission, and plasticity in the brain. Accumulating evidence suggests that neuronal PLC is critically involved in multiple aspects of GABAergic functions. Therefore, a better understanding of mechanisms by which neuronal PLC regulates GABAergic inhibition is necessary for revealing an unrecognized linkage between PLC and epilepsy and developing more effective treatments for epilepsy. Here we review the function of PLC in GABAergic inhibition in the brain and discuss a pathophysiological relationship between PLC and epilepsy.

## 1. Introduction

Epilepsy, one of the most common neurological disorders, is characterized by repeated spontaneous seizures with abnormal hyperexcitation and synchronous discharge of neurons [1]. Approximately 60 million people worldwide suffer from epilepsy with cognitive and psychiatric comorbidities [2,3]. Although several biological factors have been identified as an etiology of epilepsy, including genetic mutation, brain injury, tumor, and aging, the precise cause of epilepsy in most cases is still unknown [4,5]. One of the hypotheses explaining the pathophysiological mechanism of epilepsy is that the disruption of excitation and inhibition balance (E/I balance) could generally lead to abnormal excitability of neurons [6,7,8]. In the brain, neurons receive numerous excitatory and inhibitory synaptic inputs and once the synaptic potentials in dendrites and soma are integrated together, neurons produce axon potentials with various shapes, rates, and patterns of firing [9]. E/I balance either by increasing excitation or decreasing inhibition is associated with the hyperexcitation of neurons, which can cause epileptic seizures [7,10,11].

γ-aminobutyric acid (GABA) is a major inhibitory neurotransmitter in the brain and using GABA, GABAergic inhibitory neurons primarily regulate the excitability of neurons. GABAergic neurons produce GABA from glutamate using glutamic acid decarboxylase (GAD) and this synthesized GABA is packaged into synaptic vesicles at synaptic terminals through vesicular GABA transporters (VGATs). Synaptically released GABA binds to both presynaptic and postsynaptic GABA receptors (GABA_A_ and GABA_B_) and suppresses the excitation of presynaptic terminals and postsynaptic neurons. In addition, the uptake of released GABA at GABAergic synapses is mediated by GABA transporters (GATs). Molecular and cellular abnormalities of GABA synthesis, release, uptake, and GABA receptor-mediated signaling can alter E/I balance in neurons and the dysfunctions of any of these processes may be implicated in neurological disorders including epilepsy, schizophrenia, and autism [12,13,14]. As a matter of fact, the therapeutic rationale of the majority of current antiepileptic drugs (AEDs) is to restore altered E/I balance by elevating the level of GABA at synapses and potentiating the functions of GABA receptors. Hence, a better understanding of the underlying molecular mechanisms that regulate GABAergic inhibition in the brain will be crucial to identify new drug targets, as well as to increase the efficacy and minimize the side effects of antiepileptic drugs.

**Figure 1:**
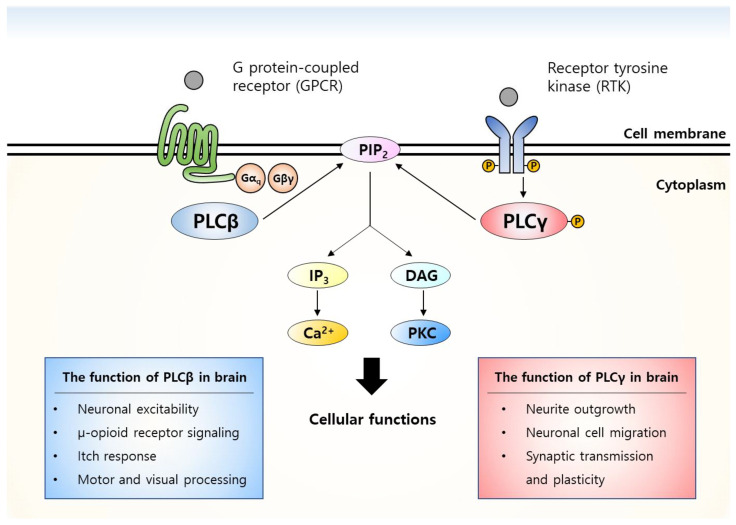
The principal PLC signaling cascades and functions in the brain. External ligands such as neurotransmitters and neurotrophic factors bind to and activate the upstream transmembrane receptors of PLC. PLCβ is activated by Gα_q_ and Gβγ subunits of G protein-coupled receptors (GPCRs), whereas the activation of PLCγ is triggered by the phosphorylation of receptor tyrosine kinases (RTKs). Activation of PLC hydrolyzes phospholipid PIP_2_ into IP_3_ and DAG and these second messengers mediate diverse neuronal functions.

Phospholipase C (PLC) is an essential enzyme in intracellular signal transduction cascades (Figure 1). PLC hydrolyzes phosphatidylinositol 4,5-bisphosphate (PIP_2_), generating secondary signal transducers including inositol 1,4,5-triphosphate (IP_3_) and diacylglycerol (DAG). IP_3_ increases intracellular calcium level by binding to IP_3_ receptors in the endoplasmic reticulum and DAG activates protein kinase C (PKC)-related signaling cascades. Throughout the body, PLC is associated with key cellular processes such as proliferation, differentiation, migration, and survival [15,16,17,18]. There are in total 13 mammalian isozymes of PLC including β (1–4), γ (1, 2), δ (1, 3, 4), ε, ζ, and η (1, 2), which are classified according to their distinct domain structures and biochemical properties. Each PLC isozyme is differentially expressed among tissues and regulates the complex cellular functions in a tissue-dependent manner. Among these PLC isozymes, PLCβ and PLCγ are major PLC enzymes abundantly expressed in the brain and play diverse roles in neuronal functions. In this review article, we summarize the molecular and cellular mechanisms of GABAergic inhibition on the regulation of E/I balance. Then we particularly focus on the role of PLC in GABAergic inhibition. Finally, we discuss the potential relationship between PLC and epilepsy.

## 2. Epilepsy Model and Antiepileptic Drugs

There are several epilepsy animal models to study the mechanism of epileptogenesis and evaluate the efficacy of AEDs [19,20,21,22,23] (Table 1). Animal models of epilepsy share similar pathological mechanisms as well as mimic the seizure behaviors of human patients. Therefore, drug screening using various epilepsy models is useful and essential to develop new AEDs. Chemoconvulsants such as pilocarpine and kainate (also referred as kainic acid, KA) are generally used to generate acute seizures within a relatively short period of time by systemic- or micro-injection. Pilocarpine is a muscarinic acetylcholine receptor agonist and pilocarpine-induced seizure has been well established as an epilepsy model. Systemic injection of pilocarpine induces tonic–clonic generalized seizures by activating M1 muscarinic receptors, especially in hippocampal neurons, and causes electrophysiological and morphological changes in the hippocampus [24,25,26]. KA is an L-glutamate analog and activates ionotropic KA receptors that are usually expressed in both excitatory and inhibitory synapses. Administration of KA causes neuronal depolarization and excitotoxic damage, particularly in the hippocampus, eventually leading to spontaneous seizures [27,28,29]. In another acute epilepsy model, electrical stimulation, so-called electrical kindling, is repeatedly delivered to the specific brain regions such as the hippocampus and amygdala. This electrical kindling increases NMDA receptor-dependent synaptic transmission, neuronal loss, and mossy fiber sprouting in the hippocampus, all of which are similar to the deficits in human epilepsy patients [30,31,32].

Unlike the acute seizures by chemical stimulants or electrical stimulation, genetic models of epilepsy in rats and mice can provide more direct insight into the genetic etiology of human epilepsy. The Genetic Absence Epilepsy Rat from Strasbourg (GAERS) is an absence seizure model that is characterized by a brief and nonconvulsive behavioral arrest and apparent unconsciousness with spike-and-wave discharges (SWDs) on electroencephalographic recordings [33]. The Wistar Albino Glaxo from Rijswijk (WAG/Rij) is also one of the absence seizure models, while the specific pathophysiological mechanism of these inbred strains is still not fully understood. Dilute brown agouti coat color (DBA/2) mice frequently show tonic-clonic seizures in response to a specific auditory stimulus [34,35]. Similarly, genetically epilepsy-prone rats (GEPR) exhibit sound-induced seizures with GABAergic, serotonergic, and noradrenergic deficits [36,37,38,39,40].

Currently, many AEDs have been developed for the treatment of complex seizure types [44]. Mechanisms of action of major AEDs are to decrease neuronal excitation by controlling voltage-gated ion channels and glutamatergic neurotransmission or to increase neuronal inhibition by upregulating GABA level and potentiating the responsiveness of GABA receptors. In this review, we will focus on the AEDs targeting GABAergic mechanisms (Table 2). Both benzodiazepines and barbiturates are positive allosteric modulators of GABA_A_ receptors and act on GABA_A_ receptors by increasing the conductance of chloride through ion channels [45,46,47], yet these drugs regulate GABA_A_ receptors in different ways. Benzodiazepines bind to GABA_A_ receptors only when in the presence of GABA, while barbiturates, when at high concentrations, can bind to GABA_A_ receptors even without ambient GABA. Phenobarbital, an allosteric modulator of GABA_A_ receptors, has been widely used for the treatment of status epilepticus and generalized tonic-clonic seizures. Upon binding to GABA_A_ receptor subunits, it increases the influx of Cl^–^ into neurons and therefore reinforces the hyperpolarization, causing the inhibition of neuronal excitation [44,46,48]. Although effective in reducing seizures, phenobarbital has severe cognitive and behavioral adverse effects, including decreased consciousness, dizziness, nystagmus, and ataxia [49]. Vigabatrin, an irreversible inhibitor of mitochondrial enzyme GABA transaminase, blocks the catabolic process of GABA [50,51]. Tiagabine is a selective competitive inhibitor of GABA transporter GAT-1, which blocks the reuptake of GABA in the synaptic cleft [52]. Vigabatrin and tiagabine increase the level of ambient GABA at synapses so that they can facilitate GABAergic inhibition.

## 3. GABAergic Dysfunction in Epilepsy

Excitatory and inhibitory synaptic currents precisely coordinate neuronal functions at the level of the synapse and neural circuit. E/I balance in the brain is determined by several physiological factors. For instance, synapse development, transmission, and plasticity modulate E/I balance at the synapse level, while the firing properties of neurons and spatiotemporal synchronization of neuronal firing can determine E/I balance at the level of the neural circuit [66,67]. At the most fundamental level, however, E/I balance is governed by neurotransmitters glutamate and GABA, both of which are primary neurotransmitters to regulate E/I balance in the brain. The concentration of glutamate in the mammalian brain is approximately 80–100 nmol/mg protein and that of GABA is 10–30 nmol/mg protein [68]. Although the population of GABAergic neurons is also much smaller than that of excitatory neurons, which is about 25–30% of excitatory neurons, GABAergic neurons strongly control the excitation of neurons with local and long-distance innervations in the brain [69]. Previous studies have observed altered GABAergic inhibition in both epilepsy animal models and human patients. In temporal lobe epilepsy patients as well as in the pilocarpine-induced epilepsy animal model, mRNA and protein levels of GAD65 and GAD67 were markedly increased in the hippocampus, probably to facilitate GABA synthesis and protect against the long-term hyperexcitability of neurons [70,71]. Two isoforms of GAD, GAD65, and GAD67, are expressed in distinct subcellular locations during neuronal development. GAD67 is mostly distributed in neuronal cell bodies, whereas GAD65 is highly expressed in axon terminals [72]. GAD65 knockout mice showed increased seizure susceptibility and the genetic deletion of GAD67 caused severe developmental defects and early deaths in mice [70,73,74,75,76]. On the other hand, elevated GAD67 expression in the hippocampal CA3 region by using recombinant adeno-associated virus (AAV) significantly decreased the seizure generation in temporal lobe epilepsy models [77]. These results indicate that the manipulation of GABA level by targeting the GAD enzyme can be a therapeutic strategy for maintaining appropriate excitability of neurons and treating epilepsy [77]. VGAT is also responsible for synaptic inhibition at GABAergic inhibitory synapses and its dysfunction is related to epilepsy. However, the specific role and contribution of VGAT is still debated in epilepsy animal models. For instance, VGAT level was found to decrease in animal epilepsy models such as cortical dysplasia, Mongolian gerbil, and picrotoxin model. Yet other studies, in contrast, reported no change of VGAT in the kainic acid model or increased VGAT expression in the pilocarpine model [78,79,80,81,82,83,84,85]. Mutation of GAT-1 was identified in epileptic encephalopathy patients, resulting in an impaired GABA reuptake process [86]. In addition to neuronal GAT-1, astrocytic GAT-1 is also crucial to GABAergic inhibition in neural circuits and its currents were attenuated in the absence seizure model [87]. This evidence supports the idea that GABA synthesis and reuptake are essential for maintaining GABAergic inhibition and the dysregulation of these processes can induce epileptic seizures.

Activation of GABA receptors by GABA binding initiates and critically mediates the postsynaptic effects of GABAergic synaptic transmission. There are two types of GABA receptors in the brain, which are GABA_A_ receptors and GABA_B_ receptors. GABA_A_ receptors are ligand-gated ion channels and function to mediate the majority of fast inhibitory synaptic transmission in the brain. Activation of GABA_A_ receptors leads to the hyperpolarization of neurons by increasing the movement of Cl^–^ into the cytosol, which prevents the action potential for neuronal activity [88,89,90]. In humans, GABA_A_ receptors that consist of five subunits, have 19 distinct subunits including α (1–6), β (1–3), γ (1–3), δ, ε, θ, π, and ρ (1–3). Unlike GABA_A_ receptors, GABA_B_ receptors are G protein-coupled receptors (GPCRs) and are responsible for the slow inhibitory function. They mediate the hyperpolarization of neurons by opening inwardly-rectifying K^+^ channels and inactivating the voltage-gated Ca^2+^ channels [91,92]. Functional GABA_B_ receptors are assembled from two subunits, GABA_B1_ and GABA_B2_, to form heterodimers [93]. Any dysfunction of GABA receptor-mediated signaling is directly implicated in various neurological disorders and psychiatric disorders [94,95]. Importantly, genetic and functional alterations of GABA receptors are closely linked to epilepsy. Mutations of GABA receptors have been identified in human epilepsy patients. For example, mutations in the α1 subunit of the GABA_A_ receptor were observed in human juvenile myoclonic epilepsy [96]. Missense variants of α1, α5 subunits in GABA_A_ receptors were found in early-onset epilepsy patients [97]. Moreover, the γ2 subunit of the GABA_A_ receptor was mutated in children with absence seizures [98]. Consistent with these human studies, genetic ablation of GABA_A_ receptors in mice led to epileptic seizures [99]. The expression of GABA_A_ receptors was also reduced in the rodent hippocampus following pilocarpine or kainic acid-induced seizures [100,101]. As with GABA_A_ receptors, the mutations of GABA_B_ receptors have been reported in epilepsy. Polymorphisms of the GABA_B_ receptor were frequently present in temporal lobe epilepsy patients [102,103]. In animal studies, the expression of the GABA_B_ receptor was decreased in the rat epilepsy model [104]. GABA_B_ receptor knockout mice showed spontaneous seizures with hyperalgesia, hyperlocomotion, and memory deficit [105]. In current antiepileptic treatments, some antiepileptic drugs enhance GABAergic inhibition through the elevation of brain GABA levels or the potentiation of GABA_A_ receptors [46,47,106]. As mentioned above, barbiturates and benzodiazepines allosterically modulate GABA_A_ receptors and are widely used as antiepileptic drugs [107,108]. Conversely, GABA_A_ receptor antagonists including bicuculline, picrotoxin, and pentylenetetrazol induce seizures and are used to experimentally model epilepsy [109]. Together, a deeper understanding of the GABAergic system and GABA receptor-mediated signaling in the brain is vital to the identification of etiology and therapeutic targets for epilepsy.

## 4. Phospholipase C Beta (PLCβ) and GABAergic Inhibition

PLCβ is activated by Gα_q_ and Gβγ subunits of G protein-coupled receptors (GPCRs) and interacts with diverse GPCRs such as 5-HT receptors, metabotropic glutamate receptors, and muscarinic acetylcholine receptors [110,111,112]. PLCβ is abundantly expressed in the brain and plays a pivotal role in regulating neuronal activity. Several studies have investigated the role of PLCβ in neuronal cells by examining PLCβ isoform-specific knockout mice. PLCβ1 knockout mice displayed an altered anxiety level and memory impairment [113]. When PLCβ3 was genetically deleted in mice, these PLCβ3-deficient mice showed high sensitivity to an opioid agonist, indicating that PLCβ3 would be a key regulator in opioid signaling and addiction [114]. PLCβ4 knockout mice exhibited deficits in visual processing, nociceptive responses, muscular coordination, and synapse elimination in the cerebellum [115,116,117].

**Figure 2:**
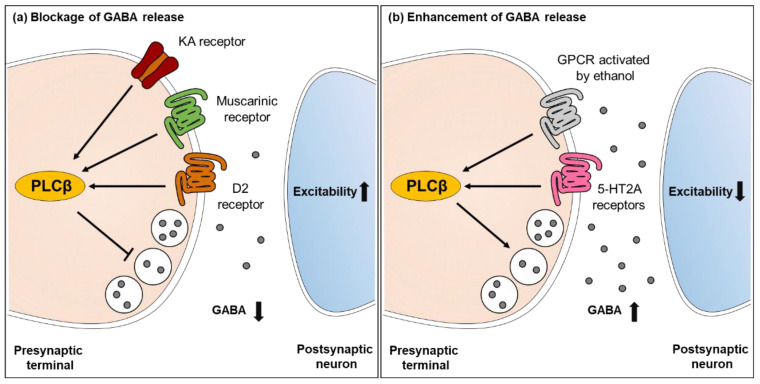
The functions of PLCβ in GABAergic inhibition. (**a**) PLCβ is activated by diverse GPCRs such as KA receptor, muscarinic receptor, and D2 receptor. Activation of PLCβ suppresses presynaptic GABA release, resulting in increased excitation of postsynaptic neurons. (**b**) However, in other neuronal types, activation of PLCβ by ethanol or serotonin increases presynaptic GABA release and consequently decreases the excitation of postsynaptic neurons.

In addition, a large number of studies have demonstrated that PLCβ is implicated in regulating GABAergic inhibition. Acetylcholine can regulate GABAergic synaptic transmission by affecting hyperpolarization, depolarization, GABA release, and oscillatory properties of GABAergic interneurons in the hippocampus [118]. Acetylcholine binds to and activates two types of receptors, which are nicotinic acetylcholine receptor (nAChR, ligand-gated ion channel) and muscarinic acetylcholine receptor (mAChR, metabotropic receptor). mAChR is a GPCR and is classified into five subtypes. Among these five subtypes, M1, M3, and M5 receptors are coupled with the Gα_q_ protein and can activate the PLCβ signaling pathway. Notably, the activation of muscarinic receptors is associated with the generation of epileptic seizures [119]. Activation of muscarinic M1 and M2 receptors by muscarinic agonists muscarine and oxotremorine suppressed GABA release, reducing the amplitude of evoked inhibitory postsynaptic currents (eIPSCs) in the rat auditory cortex [120]. On the contrary, the application of PLC blocker U73122 alleviated the oxotremorine-induced depression of eIPSC amplitude. This result clearly shows that the activation of muscarinic acetylcholine receptors can inhibit GABA transmission via the PLCβ pathway, eventually altering neuronal excitation. In addition, KA receptors are implicated in epilepsy and KA-induced seizures. Interestingly, KA receptors can affect GABAergic synaptic transmission in the presynaptic terminal. In the hippocampus, the activation of KA receptors attenuated GABA release through the PLCβ pathway in immature CA3 neurons of rats [121,122]. Moreover, presynaptic D2 receptors activated the PLC-IP_3_-calcineurin signaling cascade and decreased GABA release at striatopallidal terminals of medium-sized spiny GABAergic neurons [123]. These results reveal that several neurotransmitters act on presynaptic receptors and can activate presynaptic PLCβ. Once activated, PLCβ stimulates the downstream signaling pathway at the GABAergic presynaptic terminal, leading to the inhibition of GABA release. On the other hand, it is also likely that PLCβ is involved in enhancing GABA release. In the rat cerebellum, ethanol increased spontaneous GABA release from Purkinje cells through PLC activation, and the suppression of PLC by using PLC antagonist edelfosine prevented the ethanol-induced GABA release [124]. Similarly, 5-HT2A receptors activated PLCβ and facilitated GABA release at the thalamic reticular presynaptic terminal [125,126]. From this evidence, it appears that PLC and the downstream signaling pathway mainly suppress GABA release at the GABAergic presynaptic terminal, while they also enhance GABA release depending on cell types and interacting receptors (Figure 2).

PLCβ and its relevance to epilepsy have been strongly suggested in many studies (Table 3). Genetic mutations of PLCβ1 were found in human infantile epileptic encephalopathy [127,128,129,130]. In animal studies, PLCβ1 knockout mice exhibited status epilepticus with generalized and tonic-clonic seizures and prematurely died starting from three-weeks-old [131]. Moreover, the population of somatostatin interneurons that primarily target the distal dendrite of a neuron was reduced in the hilus of PLCβ1 knockout mice. Loss of PLCβ1 function by using transgene insertion in mice caused late-onset epileptic symptoms at six months of age with aberrant mossy fiber sprouting in the hippocampus, decreased NMDA receptor density in the stratum oriens of the CA1, and increased apoptosis [132]. In the thalamus, dysfunction of PLCβ4 in thalamocortical neurons induced abnormal burst firing, resulting in absence seizures characterized by spike-wave discharges on electroencephalography (EEG) [133]. On the other hand, the expression of PLCβ was remarkably increased in the hippocampus in the chemical-induced epilepsy model, possibly to protect against neuronal damage [134,135,136]. Furthermore, both PLCβ1 and PLCβ4 expression in hippocampal GABAergic interneurons was reduced following pilocarpine-induced seizures [136]. Collectively, these findings suggest that the function of PLCβ is potentially important for preventing excessive neuronal excitability in epilepsy.

## 5. Phospholipase C Gamma (PLCγ) and GABAergic Inhibition

PLCγ is usually activated by the phosphorylation of receptor tyrosine kinases (RTKs) in response to extracellular ligands such as brain-derived neurotrophic factor (BDNF), epidermal growth factor (EGF), platelet-derived growth factor (PDGF), nerve growth factor (NGF), and fibroblast growth factor (FGF). PLCγ has Src homology (SH) domains such as SH2 and SH3. SH2 binds to the tyrosine-autophosphorylation site of RTKs and SH3 interacts with other signaling molecules including the c-Cbl family of E3 ubiquitin ligase and proline-rich motifs on SOS1 which is a guanine nucleotide exchange factor [141,142]. Two isoforms of PLCγ, PLCγ1, and PLCγ2, exhibit distinct expression patterns throughout the body. PLCγ1 is highly expressed in diverse tissues including the brain, while PLCγ2 is primarily enriched in immune cells and its expression in the nervous system is relatively weak [18,143]. Polymorphism in PLCγ1 was frequently found in bipolar patients [144,145] and excitatory neuron-specific PLCγ1 knockout mice exhibited manic-like behaviors such as hyperlocomotion, decreased anxiety, increased hedonic action, and impaired learning and memory performance [146]. Moreover, the ablation of PLCγ1 in neuronal progenitor cells resulted in impaired axon guidance during developmental stages [147].

Meanwhile, it was found that the treatment of BDNF suppressed GAT-1-mediated GABA uptake and this inhibition was abolished by TrkB and PLC inhibitors such as K252a and U73122 in rat hippocampal nerve terminals [148]. In another study, the activation of PLC did not change GABA uptake in neurons, but decreased GABA uptake in astrocytes [149]. These findings suggest that PLC can control GABAergic transmission by modulating GABA reuptake in a cell type-dependent manner. Many studies have focused on the function of the BDNF/TrkB/PLCγ pathway in controlling GABA_A_ receptor signaling. The physiological role of BDNF in epilepsy has been well documented, but it is still unclear whether the effect of BDNF is facilitating or inhibiting epileptic seizures [150,151]. It has been shown that short-term and long-term treatment of BDNF may exert differential effects on GABAergic transmission. Acute BDNF treatment decreased both evoked and spontaneous inhibitory postsynaptic currents (IPSCs), which was caused partly by a rapid reduction in postsynaptic GABA_A_ receptor number, but did not affect excitatory postsynaptic currents (EPSCs) in hippocampal CA1 neurons. Furthermore, BDNF-induced attenuation of IPSCs was significantly suppressed by TrkB inhibitor and PLC inhibitor, indicating that these effects by BDNF seem to be mediated by TrkB and PLC [152,153]. In the mouse cerebellum, acute application of BDNF also reduced postsynaptic GABA response in cerebellar granule cells, whereas the same BDNF treatment potentiated GABA_A_ receptor functions via the TrkB-PLCγ pathway in cerebellar Purkinje cells and consequently enhanced the amplitude of mIPSCs [154]. Another study showed that BDNF promoted the maturation of GABAergic neurons and upregulated the expression of GABA_A_ receptor in cultured hippocampal neurons. Given the evidence above, although direct evidence was lacking in this report, this trophic effect of BDNF might be potentially mediated by PLC [155]. Interestingly, the effect of the BDNF/TrkB/PLC signaling pathway on GABA_A_ receptor function can change across the developmental stages. It was shown that BDNF treatment acutely potentiated postsynaptic GABA_A_ receptor-mediated currents in the rat hippocampus at postnatal day six. However, through TrkB-PLCγ signaling, BDNF later induced a long-lasting inhibition of postsynaptic GABA_A_ receptor at postnatal day 14 [156]. In addition, the BDNF-TrkB-PLCγ pathway rapidly increased the number of GABA_A_ receptors in the developing rat visual cortex [157], demonstrating that PLC may be one of the critical players regulating GABA_A_ receptor expression, function, and eventually GABAergic inhibition.

In line with the above findings, it appears that PLC is also implicated in GABA_B_ receptor-mediated functions. GABA_B_ receptors are located in both presynaptic and postsynaptic neurons. Presynaptic GABA_B_ receptors inhibit adenylyl cyclase and Ca^2+^ channels through Gα_i/o_ and Gβγ proteins, respectively, thus inhibiting additional GABA release. Upon activation, postsynaptic GABA_B_ receptors function to open K^+^ channels and cause the hyperpolarization of postsynaptic neurons, so that it suppresses neuronal excitation. GABA_B_ receptors can also stimulate PLC and induce BDNF secretion to facilitate the maturation of GABAergic synapses at developmental stages [158]. Released BDNF increased the expression of GABA_A_ receptor β2, β3 subunits in the postsynaptic neuronal surface, and enhanced GABAergic response [159]. As further evidence showing the functional connection between the GABA_B_ receptor and PLC, baclofen is a GABA_B_ receptor agonist and the application of PLC inhibitor U73122 suppressed GABA_B_ receptor-mediated Ca^2+^ increase by baclofen in human retinal pigment epithelium [160]. Moreover, GABA_B_ receptor activation could modulate the insulin-like growth factor 1 signaling pathway via PLC activation to prevent apoptosis in cerebellar granule neurons [161].

**Figure 3:**
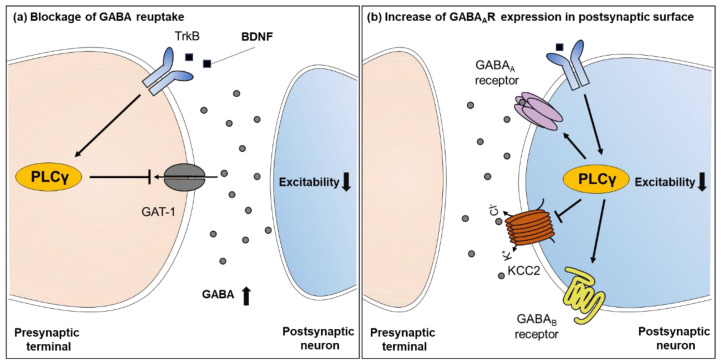
The functions of PLCγ in GABAergic inhibition. (**a**) Activation of PLCγ by the BDNF/TrkB pathway prevents GABA reuptake by GAT-1, leading to the accumulation of extracellular GABA. (**b**) Activation of PLCγ increases the surface expression of GABA_A_ receptors. In addition, PLCγ inhibits Cl^–^ extrusion via the regulation of KCC2 function. Activation of GABA_B_ receptors induces BDNF secretion through PLCγ activation and consequently increases the expression of GABA_A_ receptors in the postsynaptic membrane, therefore decreasing the excitation of neurons.

Neuronal K^+^-Cl^–^ cotransporter KCC2 mediates Cl^–^ extrusion from the cytosol to maintain a low intracellular concentration of Cl^–^ and hyperpolarize postsynaptic neurons [162,163,164]. The mRNA and protein expression of KCC2 were decreased in the hippocampus following kindling-induced seizures. In addition, both KCC2 mutation and downregulated KCC2 expression were identified in epilepsy patients [165]. Importantly, BDNF application could decrease the expression of KCC2 in hippocampal neurons, but the mutation of the PLCγ interaction site in TrkB rather increased the expression of KCC2 protein by exposure to BDNF [166]. Altogether, this evidence clearly indicates that PLCγ plays a key role in the regulation of GABAergic inhibition by exerting diverse actions on both presynaptic and postsynaptic sites (Figure 3).

Most studies of PLCγ on epilepsy have focused on the BDNF/TrkB/PLCγ1 signaling cascade in epileptogenesis. It was shown that seizures induced by electrical stimulation were significantly attenuated by genetic knockout of TrkB and PLCγ1 [138,167]. In addition, blocking PLCγ1 binding to TrkB suppressed epileptogenesis in the hippocampus [137,139], implying that BDNF/TrkB/PLCγ1 signaling may contribute to the etiology of epileptogenesis by shifting E/I balance towards more excitation. However, a recent study by our group found that GABAergic neuron-specific PLCγ1 knockout mice over six months of age exhibited late-onset seizures and other behavioral abnormalities owing to impaired GABAergic inhibition in the hippocampus [140]. In spite of many unanswered questions, these studies highlight a critical role for PLC in the generation of epilepsy, while its exact role may be dependent on neuronal cell types and brain regions.

## 6. Concluding Remark and Future Perspectives

Although epilepsy is one of the most common neurological disorders, it is apparent that the treatment of epilepsy still remains less than optimum. Many AEDs have been developed over the past decades, most of which were effective in treating epileptic seizures. However, 30–40% of epilepsy patients are not adequately controlled by current AEDs and these patients develop refractory epilepsy [168]. An alternative treatment option is brain surgery that partially removes the epileptic brain region, but it inherently has a high-risk with serious side effects. As such, a better understanding of the fundamental etiology, as well as pathophysiological mechanism of epilepsy, is crucial to identify novel and more effective treatments. Although much needs to be further investigated, a recent hypothesis of E/I imbalance in the etiology of epilepsy advanced our understanding of the molecular, cellular, and synaptic mechanisms underlying epilepsy. Most notably, it has been demonstrated that despite their small population, GABAergic neurons and GABAergic inhibition have a powerful effect in maintaining optimal E/I balance. In this review, we briefly described the potential and critical role of PLC in regulating GABAergic inhibition and epilepsy in the brain. As documented throughout this review, it is increasingly evident that PLC plays an essential role in controlling GABAergic transmission at both presynaptic and postsynaptic sites, through modulating GABA release, reuptake, and GABA receptor-mediated signaling. In addition, evidence is accumulating that major PLC enzymes highly expressed in the brain, including PLCβ and PLCγ, are directly and indirectly linked to epileptogenesis both in human patients and animal models. As PLC can exert differential molecular actions on neurons via a cell type-specific manner, undoubtedly, further work will be required to dissect the exact role of each neuronal PLC isoform on regulating GABAergic inhibition and E/I balance in both excitatory and inhibitory neurons. Together, PLC can be a potential and new therapeutic target for epilepsy, and pharmacological manipulation of specific PLC isoform may prove therapeutically fruitful in the treatment of epilepsy.

## Figures and Tables

**Table 1:** The animal models of epilepsy.

Type	Epilepsy Model	Mechanism	Symptoms	Reference
Genetic	Genetic Absence Epilepsy Rat from Strasbourg (GAERS)	Inbred strain Mutation of *Cacna1h* gene encoding CaV3.2 T-type calcium channel	Spike-and-wave discharges (SWD) in EEG	[33,41]
	WAG/Rij	Polygenic gene mutation	Spike-and-wave discharges (SWD) in EEG	[42,43]
	DBA/2	Mutation of *Asp2* gene	Audiogenic seizures	[34,35]
	Genetically epilepsy-prone rats (GEPR)	GABAergic, serotonergic, noradrenergic deficits	Audiogenic, generalized tonic-clonic seizures	[33]
Electrical	Kindling	Lower threshold by repeated stimulation	Temporal lobe epilepsy	[30,31,32]
Chemical	Pilocarpine	Muscarinic acetylcholine receptor agonist	Generalized tonic–clonic seizures	[24,25,26]
	Kainic acid	L-glutamate analog	Temporal lobe epilepsy	[27,28,29]

**Table 2:** Current antiepileptic drugs with GABAergic effects.

Drug	Mechanism	Epilepsy Types	Reference
Potassium bromide	GABA potentiation	Generalized tonic-clonic seizures, myoclonic seizures	[53,54]
Phenobarbital	Potentiation of GABA_A_ receptor	Partial and generalized convulsive seizures	[55]
Primidone	GABA potentiation	Partial and generalized convulsive seizures	[56]
Diazepam	Potentiation of GABA_A_ receptor	Status epilepticus	[57,58,59]
Valproate	Multiple mechanisms with glutamate inhibition, blockade of sodium and T-type calcium channels, inhibition of GABA transaminase and re-uptake	Partial and generalized convulsive seizures, absence seizures	[60,61]
Clonazepam	Potentiation of GABA_A_ receptor	Juvenile myoclonic epilepsy	[62,63]
Benzodiazepines	Potentiation of GABA_A_ receptor	Partial and generalized convulsive seizures, Lennox–Gastaut syndrome, myoclonic seizures	[58]
Vigabatrin	Inhibition of GABA transaminase	Infantile spasms, complex partial seizures	[64,65]
Tiagabine	Inhibition of GABA transporter	Partial seizures	[52]

**Table 3:** The functions of PLC in epilepsy.

PLC Isozyme	Animal or Human Study	Phenotype	Reference
PLCβ1	Genetic knockout mice	Early-onset epileptic encephalopathy	[127]
	Mongolian gerbils mice	Increased PLCβ1 expression after seizures	[135]
	Genetic knockout mice	Malignant migrating partial seizures in infancy	[128]
	Pilocarpine-induced status epilepticus in mice	Decreased PLCβ1 expression in hippocampal interneurons after seizures	[136]
	Homozygous deletions or nonsense variant in human	Infantile epileptic encephalopathy	[130]
PLCβ4	Genetic knockout mice	Absence seizures	[133]
PLCγ1	TrkB mutation mice in PLCγ1 binding domain	Decreased pilocarpine-induced status epilepticus	[137]
	Heterozygote knockout mice	Decreased kindling-induced seizures	[138,139]
	GABAergic neuron-specific knockout mice	Late-onset seizures	[140]

## Data Availability

Not applicable.

## Abbreviations

E/I balance	Excitation and inhibition balance
PLC	Phospholipase C
GABA	γ-aminobutyric acid
GAD	Glutamic acid decarboxylase
VGAT	Vesicular GABA transporter
AED	Antiepileptic drug
PIP_2_	Phosphatidylinositol 4,5-bisphosphate
IP_3_	Inositol 1,4,5-triphosphate
DAG	Diacylglycerol
PKC	Protein kinase C
GPCR	G protein-coupled receptor
RTK	Receptor tyrosine kinase
BDNF	Brain-derived neurotrophic factor
TrkB	Tropomycin receptor kinase B
AAV	Adeno-associated virus
GAT	GABA transporter
EPSC	Excitatory postsynaptic current
IPSC	Inhibitory postsynaptic current
KCC	K^+^-Cl^–^ cotransporter
nAChR	Nicotinic acetylcholine receptor
mGluR	Metabotropic glutamate receptor
EGF	Epidermal growth factor
PDGF	Platelet-derived growth factor
NGF	Nerve growth factor
FGF	Fibroblast growth factor
